# A Polarization Control System for Intensity-Resolved Guided Mode Resonance Sensors

**DOI:** 10.3390/s140305198

**Published:** 2014-03-12

**Authors:** Sheng-Fu Lin, Fu-Chen Chang, Zhi-Heng Chen, Chih-Ming Wang, Tsung-Hsun Yang, Wen-Yih Chen, Jenq-Yang Chang

**Affiliations:** 1. Department of Optics and Photonics, National Central University, Jhongli 32001, Taiwan; E-Mails: 982406004@cc.ncu.edu.tw (S.-F.L.); warcrift3@gmail.com (F.-C.C.); hanksck@yahoo.com.tw (Z.-H.C.); thyang@dop.ncu.edu.tw (T.-H.Y.); 2. Department of Opto-Electronic Engineering, National Dong Hwa University, Hualien 97401, Taiwan; E-Mail: wangcm@mail.ndhu.edu.tw; 3. Department of Chemical and Materials Engineering, National Central University, Jhongli 32001, Taiwan; E-Mail: wychen@ncu.edu.tw

**Keywords:** photonic sensors, guided mode resonance, polarization ellipse

## Abstract

In this study, a polarization-control setup for intensity-resolved guided mode resonance sensors is proposed and demonstrated experimentally. The experimental results are in good agreement with the simulation data based on rigorous coupled wave approach calculations. The proposed intensity-resolved measurement setup transfers polarization ellipses, which are produced from guided mode resonance to a linear polarization state under a buffer solution condition, and then suppresses the signals to dark using a polarization-control set. Hence, any changes in the refractive index results in an increase in the intensity signals. Furthermore, no wavelength-resolved or angular-resolved measurement is needed in this scheme. According to the experimental results, a wide linear detection range of 0.014 refractive index units is achieved and the limit of detection is 1.62E-4 RIU.

## 1. Introduction

The demand for biosensing, especially for investigations related to food safety, infectious diseases and security issues is currently increasing [1–3]. Label-free methods provide a rapid and simple detection platform for further investigation of molecular interactions [3,4]. Guided mode resonance (GMR) devices [5–7], one of the recently arising types of optical label-free sensors, have been also demonstrated to offer dependable information about biomolecular interactions and have been developed commercially by Corning, SRU Biosystems and MicroVacuum [8–10]. The GMR device utilizes the evanescent wave around the structure surface to detect adsorption of biological material onto the sensor surface (*i.e.*, changes of refractive index). As target molecules become attached to the chip surface, the boundary conditions of the evanescent waves from resonance are changed and this results in a shift of the resonance wavelength. For typical GMR sensors, the value of the shift in the resonance wavelength is considered to be a direct indicative quantity of bioassay reactions (interactions) occurring at the structure surface [11,12]. Thus, wavelength-resolved instruments have been extensively introduced in GMR sensor systems, such as spectrometers [9,13,14], wavelength tunable light sources [8,15] or angular-resolved motor stages [10]. Since the year 2000, GMR sensors using wavelength-resolved platforms have been widely employed in bioanalytical applications, including protein-protein and aptamer-protein interactions, as well as for DNA hybridization, drug discovery and cell assays [1,8,9,16,17]. However, the wavelength-resolved instruments are expensive and also need a lot of effort to achieve high throughput configurations [8,15]. Instead of applying wavelength-resolved instruments, this study manipulates the elliptical polarization states transmitted after the GMR device for the development of an intensity-resolved, low-cost sensor. In this study, a theoretical model for this scheme is explained and the experimental results are demonstrated as well. The polarization control setup is able to suppress the background signal under any buffer conditions, and the following changes in refractive index are transferred to an increase of light intensity responses, this is not only suitable for wide bioanalytical applications but also has the potential for 2D high throughput configuration.

## 2. Experimental Section

### 2.1. System Setup

**Figure 1.**
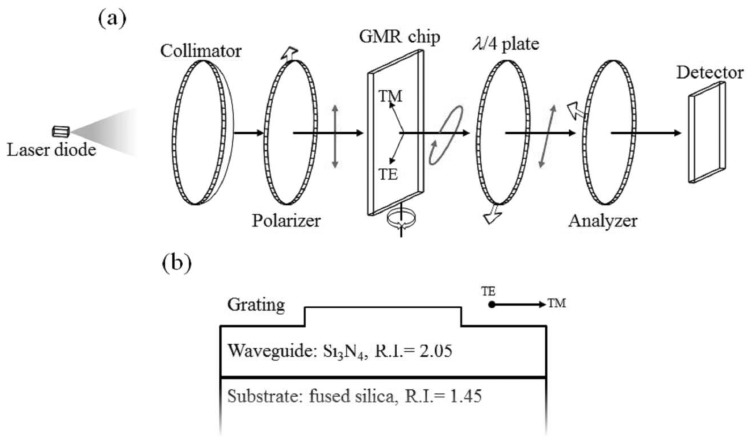
(**a**) Setup of the polarization control system. The arrows sketched on the polarizer, analyzer and quarter wave plate stand for the transmission directions and fast axis, respectively. The red arrows drawn on light path depict the polarization states of the light wave. (**b**) Sketch of surface relief GMR chip, one period is present.

Figure 1a shows the system setup. The light source used in this work is a laser diode with a working wavelength centered at 1,550 nm, purchased from Thorlabs (Newton, NJ, USA), followed by collimation optics. A linear polarizer is placed after the collimator lens to control the TE/TM ratio (TE: transverse electric; TM: transverse magnetic), which is set to 1:1 in this work. The GMR chip sealed in a homemade fluidic cell is placed on a rotational stage immediately after the first polarizer. A design for the non-reaction region is also introduced to the fluidic cell, which provides a self-reference for signal processes. The misallocation of the resonance wavelength caused by imperfection in chip fabrication can be adjusted by rotating the chip as shown in Figure 1a. In order to control the transmitted polarization state, a polarization control set composed of a quarter wave plate (λ/4 plate) is used as a compensator, with an analyzer placed right after the sealed sensing cell. All the wave plate and polarizers were purchased from Edmund Optics (Barrington, NJ, USA). Finally, there are two detectors for real-time transmission intensity capture and calibration, one for the signal and the other for reference. The detector was purchased from Gentec-EO (Lake Oswego, OR, USA). Here, a surface relief GMR chip is used in the intensity-resolved configuration to serve as an analyte-sensitive *wave plate*, which produces different amplitude TE/TM ratios and mutual phase differences (*i.e.*, different polarization ellipses). Therefore, the following quarter wave plate and analyzer set is able to transfer information about the analyte-sensitive polarization ellipses to an intensity-resolved sensor. As can be seen in Figure 1b, the GMR chip is composed of a grating and a waveguide layer on top of a substrate. The period of the grating and waveguide thickness is designed to resonant around 1,550 nm to fit the light source that is used in this work. The reason for using 1,550 nm is that all of the related passive and active devices are easily obtained. However, the GMR device could be modified to fit any possible target wavelengths.

### 2.2. Sensing Principle

**Figure 2.**
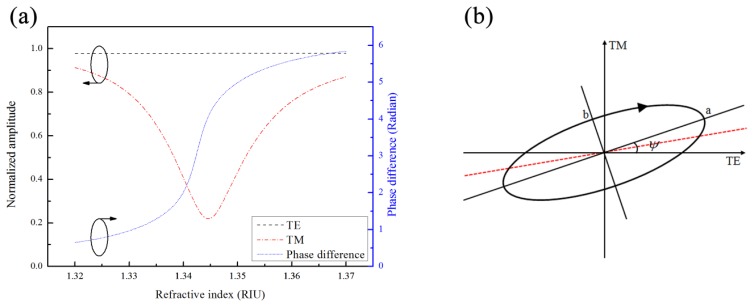
(**a**) Normalized amplitudes and mutual phase difference of light waves after GMR; (**b**) sketch of a polarization ellipse.

The GMR is a polarization dependent optical filter that couples and reflects specific wavelengths according to its geometric structure and refractive index of the surrounding environment. As the specific light (*i.e.*, resonance wavelength) illuminates the GMR structure, the grating diffracts the light into the waveguide layer at a specific angle. The resonance occurs when the diffracted light matches the propagation constant of the waveguide and results in a lower transmission spectrum [11,12,18]. Figure 2a shows the amplitude of a normalized electric field and the mutual phase difference of the TE and TM modes after the GMR device with respect to different environmental refractive indexes from 1.32 to 1.37. The data in Figure 2a are calculated by rigorous couple wave analysis (RCWA) [19]. The structure calculated here is designed to have a relatively high response when working at 1,550 nm, according to a previous study [12]. A GMR with a period of 1.21 μm, 0.25 μm thick waveguide and grating depth of 0.121 μm is calculated. The dip of amplitude in TM mode indicates the resonance condition of TM0. In contrast, the off-resonance TE mode shows no response to the variations of the refractive index, and the amplitude variation in the TE mode is less than 0.15%. On the other hand, the phase difference shows a change in behavior across the resonance dip. Since the amplitude ratio and phase difference of the two orthogonal modes across the resonance are changing, the polarization state is changing as well. It is convenient to express the general form of the polarization ellipse as follows:

(1) $$E=\left[\begin{array}{c} E_{TE}\\ E_{TM} \end{array}\right]=\left[\begin{array}{c} A\exp\left(i\delta_{TE}\right)\\ B\exp\left(i\delta_{TM}\right) \end{array}\right]\exp\left[i\left(kz-\omega t\right)\right],\delta =\delta_{TM}-\delta_{TE}$$

where *A* and *B* indicate the amplitude of the electric field from the TE and TM waves; *δ* stands for the mutual phase difference between the TE and TM waves; and *k* and *ω* denote the wave number and angular frequency. The shape parameters of the ellipse can be expressed as follows:

(2) $$a^{2}={\left[A\cos\left(\psi \right)+B\cos\left(\delta \right)\sin\left(\psi \right)\right]}^{2}+{\left[B\sin\left(\delta \right)\sin\left(\psi \right)\right]}^{2}$$

(3) $$b^{2}={\left[A\sin\left(\psi \right)-B\cos\left(\delta \right)\cos\left(\psi \right)\right]}^{2}+{\left[B\sin\left(\delta \right)\cos\left(\psi \right)\right]}^{2}$$

(4) $$\tan\left(2\psi \right)=\frac{2AB\cos\left(\delta \right)}{A^{2}-B^{2}}$$

where *ψ* is the rotational angle between the major axis and the TE axis; *a* and *b* stand for the semi axis lengths of the ellipse, as shown in Figure 2b.

The intensity-resolved GMR system with polarization control is used to monitor the changes in the polarization ellipse caused by the different refractive indexes in the sensing region. However, the polarization states must be transferred to changes in transmission intensity, which is what the detector directly responds to. Thus, a quarter wave plate and analyzer set is placed after the GMR filter. The various transmitted elliptical polarizations can be converted to a linear polarization state by the quarter wave plate whose axis is oriented parallel to either the major or minor axis of the polarization ellipse. That is, when the axis of the quarter wave plate is parallel to the “*a*” or “*b*” axis, the polarization state is transferred to the linear state, as shown by the red dashed-line in Figure 2b. Then, the direction of the linearized polarization state can be described as follows:

(5) $$\varphi =\psi +\arctan\left(1-e^{2}\right),e=\sqrt{1-{\left(\frac{b}{a}\right)}^{2}}$$

where *e* is the eccentricity of the ellipse, which depends on the length ratio of the major and minor axes. The directions of the linearized polarization are no longer equal to the *ψ* when the eccentricity is not a unit. As the polarization ellipse is linearized, an analyzer whose axis crosses the direction of the linear polarization can extinguish the transmitted light intensity. Through the polarization control set, the signal intensity can be effectively suppressed at any background refractive index (e.g., the buffer solution for different applications); therefore, subsequent changes of the polarization state due to any perturbation of the refractive index or bio-interactions result in an increase in the transmitted intensity.

### 2.3. Chip Fabrication

The surface-relief GMR chip used in this work is a high refractive index waveguide grating (Si_3_N_4_) deposited on a fused silica substrate. The high refractive index layer is deposited by plasma enhanced chemical vapor deposition (PECVD). Linear grating patterns are defined on a photo-resistant material by laser interference lithographic methods followed by reactive-ion etching to transfer a surface-relief grating to the waveguide surface. In order to demonstrate the validity of the polarization control system, a series of sodium chloride solutions with different concentrations (different refractive indexes) were introduced into the homemade fluidic cell containing the GMR chip for real-time monitoring of the transmission intensity. The refractive indexes of the solutions were also checked with a commercial refractive index meter.

## 3. Results and Discussion

**Figure 3.**
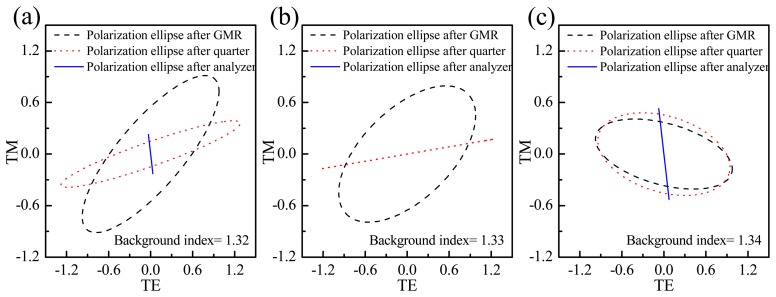
Polarization ellipses for background indexes equal to (**a**) 1.32; (**b**) 1.33; and (**c**) 1.34. The ellipse is calculated from Figure 2a; the orientations of the quarter and analyzer plates are to 34.84° and 97.97°, in order to specifically suppress the transmission intensity for the 1.33 background refractive index.

The amplitudes and the mutual phase difference information enable one to obtain the polarization ellipses transmitted for different refractive index solutions after transmission through the GMR. The dashed black circular lines in Figure 3 indicate the polarization ellipses transmitted through the GMR chip for solutions with three different refractive indexes (1.32, 1.33 and 1.34). The phase and amplitude information are the same as the data in Figure 2. Figure 3 also depicts the evolution of the polarization ellipses through the quarter wave plate and analyzer control set. The orientations of the quarter wave plate and analyzer are fixed at 34.84° and 97.97° in order to suppress the background refractive index of 1.33; however, the suppressed refractive index could be any index depending on the specific buffer solution used. The red and blue lines indicate the ellipses after the quarter wave plate and analyzer, respectively. According to the results, only the ellipse for the 1.33 condition shows a total transfer to linear polarization, extinguished by the analyzer. For the other conditions there is still a portion of intensity transmitted through the analyzer.

**Figure 4.**
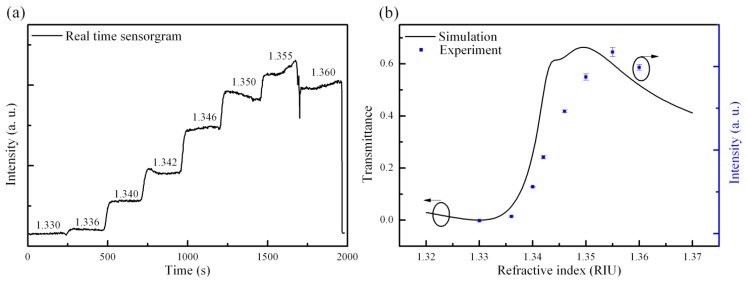
(**a**) Real-time response for 8 different refractive index solutions; (**b**) simulated and experimental intensity response; the error bar (one σ is depicted) stands for the noise level calculated from real-time results.

The grating period for the fabricated GMR chip is 1.21 μm; the thicknesses of the waveguide and grating are 0.25 μm and 0.121 μm, respectively, as measured by an ellipsometer and AFM. The GMR chip is rotated at an angle of 11.95°. Taking into account the measured structural parameters in the RCWA calculation, the amplitude and phase response at different refractive indexes are obtained. The chip meets the TM0 resonance conditions for a background index condition of 1.3445. In this study, the intensity responses after the polarization control setup are calculated using Jones calculus [20]. The suppression of the refractive index should be slightly smaller than the resonance refractive index to achieve better performance. In this work, the optimal location is designed to be at 1.33 where the TM amplitude equals 0.79. Figure 4 shows the real-time experimental results. It can be seen that there are 8 solutions with different concentrations of sodium chloride continually pumped into the fluidic cell. The intensities *versus* refractive indexes in the experimental and simulation data are also shown in the Figure 4b. Under the suppressed condition, no transmission intensity is observed, while the intensity responses of the other refractive indexes are higher than under the suppressed condition (1.33). As can be seen from the Figure 4b, a slightly broader linear response range is observed in the experimental results. This phenomenon could be contributed to by fabrication errors. In the simulation, the structural model in RCWA is perfectly extended with periodic boundaries. The simulation results from RCWA always show a much narrower resonance bandwidth than in the experimental results. As the GMR bandwidth is broadened, the intensity response is also broadened. However, the experimental results still show good agreement with the simulation results. In this study, the experimental linear response ranges from 1.336 to 1.35, which is 0.014 refractive index units (RIU). The results for the limit of detection (LOD) are strongly dependent on the noise level (3σ, standard deviation) of the whole system. The noise level significantly affects the LOD result in this work. Thus, the LOD is only 1.62E-4 RIU. Dividing the signal by a reference signal is further reduces the effects on the signal of light source fluctuations. The simulated maximum linear dynamic range of this present configuration is from 0 to 61% of the incident light intensity when the refractive index changes from 1.33 to 1.344 as shown in the Figure 4b. Thus, based on a general signal to noise (S/N) ratio of light sensitive devices, (typically around hundreds) the theoretical resolution of the refractive index that could be achieved is on the order of 10E-5 RIU; a superior resolution could be expected if any further advanced noise-reducing configurations are introduced. However, there is a trade-off between the dynamic range and resolution of refractive index. In some cases in the simulation, a large dynamic range (above 90% of the incident light intensity) is achieved. However, the operation range of refractive index is relative small. In this work, we demonstrate a large operation range configuration to further exhibit the advantages of the polarization controlled GMR. The inferior limit of the detection level obtained in this work is mainly limited by the low incident light intensity and the detector used in this setup. The light output from the laser diode is collimated to a beam with a diameter of 20 mm, which significantly decreases the light intensity illuminating on the GMR region. In such a low light condition, the S/N ratio is decreased as well. Furthermore, no advanced noise-reducing circuit is connected to the detector, which also limits the resolution of the present measurements. In order to achieve better performance, it is necessary to increase the light intensity and reduce the electronic noise.

## 4. Conclusions

In conclusion, a low-cost polarization control system for intensity-resolved GMR sensors is proposed and demonstrated. According to the changes in elliptical polarization after the GMR sensor, the polarization control set transfers the polarization ellipse into a linear state. A crossed analyzer further suppresses the transmission light intensity under buffer solution conditions. Thus, all subsequent changes of the refractive index are converted into increasing intensity responses. The polarization states are significantly changed and converted into the intensity-resolved responses. The linear range of 0.014 RIU proposed in this article is wider than that reported for intensity-resolved SPR sensors, typically around 0.002–0.007 RIU [21,22]. Thus, the linear range achieved in this work is more than sufficient for most bioanalytical applications. On the other hand, the LOD is 1.62E-4 RIU. Although the LOD reported here is inferior for further advance applications, this study demonstrates the feasibility of the concept for low-cost intensity-resolving through the use of polarization control schemes. Furthermore, this low-cost setup is practical and suitable to extend to a 2D image array for high throughput applications by replacing the detector with an image sensor.
